# Silver-coated versus uncoated locking plates in subjects with fractures of the distal tibia: a randomized, subject and observer-blinded, multi-center non-inferiority study

**DOI:** 10.1186/s13063-022-06919-0

**Published:** 2022-12-01

**Authors:** S. Schoder, M. Lafuente, V. Alt

**Affiliations:** 1 aap Implantate AG, Lorenzweg 5, 12099 Berlin, Germany; 2 Universitätsklinikum Regensburg, Klinik und Poliklinik für Unfallchirurgie, Franz-Josef-Strauß-Allee 111, 93042 Regensburg, Germany

**Keywords:** Silver-coating, Antibacterial, Tibia, Fracture, Randomized, Orthopedic, Antimicrobial

## Abstract

**Background:**

Antimicrobial coatings of implants are of interest to reduce infection rate in orthopedic surgery. Demonstration of clinical effectiveness of such coated implants to obtain market approval is challenging. The objective of this article is to define a design for a randomized controlled trial to evaluate the clinical performance of a silver-coating for locking plates for fracture treatment.

**Methods:**

The study design has to respect different criteria, such as feasibility, focus on overall complications, such as functional impairment, fracture healing, and particularly on infection rates. Distal tibia fractures were chosen due to the high prevalence of infections in this type of injuries, which warrants a particular benefit of antimicrobial prophylaxis and thus might allow to see a statistical trend in favor of the coated product. The study design was defined as a randomized, controlled, subject and observer-blinded, multi-center study in subjects with fractures of the distal tibia with a total of 226 patients. A number of 113 patients are planned for each of the two treatment arms with treatment of the fracture with a silver-coated device (first arm) or with an uncoated device (second arm). Inclusion criteria are closed fractures of the distal tibia according to the Tscherne-Oestern classification or open fractures of the distal tibia according to the Gustilo-Anderson classification in subjects older than 18 years. Primary outcome parameter is the Anticipated Adverse Device Effects (AADE) including all typical complications of this type of injury, such as functional impairment of the affected limb, non-union, and infections based on a non-inferiority study design. Also, silver-typical complications, such as argyria, are included. Secondary parameters are infection rates and fracture healing. Follow-up of patients includes five visits with clinical and X-ray evaluations with a follow-up time of 12 months.

**Discussion:**

Demonstration of clinical effectiveness of antimicrobial coatings of fracture fixation devices remains a challenge. Definition of a prospective randomized pre-market trial design and recruitment of clinical sites for such a study is possible. A confirmative proof of the expected clinical benefit in terms of reduction of device-related infections will be addressed with a prospective post-market clinical follow-up study in a second step due to the large sample size required.

**Trial registration:**

ClinicalTrials.gov NCT05260463. Registered on 02 March 2022.

## Administrative information

Note: the numbers in curly brackets in this protocol refer to SPIRIT checklist item numbers. The order of the items has been modified to group similar items (see http://www.equator-network.org/reporting-guidelines/spirit-2013-statement-defining-standard-protocol-items-for-clinical-trials/).

**Table:** 

Title {1}	Silver-coated versus uncoated locking plates in subjects with fractures of the distal tibia: a randomized, subject and observer-blinded, multi-center non-inferiority study
Trial registration {2a and 2b}	NCT05260463 [ClinicalTrials.gov] registered on 02.MAR.2022
Protocol version {3}	25.OCT.2021 Version 4.0
Funding {4}	This clinical evaluation is funded by *aap* Implantate AG and the German Federal Ministry of Education and Research (BMBF) as part of the program "Transferring medical solutions into patient care - Proving clinical evidence without delay" (funding codes: 13GW0313A, 13GW0313B, 13GW0449A and 13GW0449B). The BMBF has no role in designing the study, collection, analysis, or interpretation of the data.
Author details {5a}	Prof. Dr. Dr. Volker Alt: University Medical Centre Regensburg, Germany Dr. Stefan Schoder: *aap* Implantate AG, Berlin, Germany Michel Lafuente: *aap* Implantate AG, Berlin, Germany
Name and contact information for the trial sponsor {5b}	Dr. Stefan Schoder s.schoder@aap.de *aap* Implantate AG, Lorenzweg 5, 12099 Berlin, Germany
Role of sponsor {5c}	The sponsor is the manufacturer of the investigational device

## Introduction

### Background and rationale {6a}

Implant-related musculoskeletal infections have a severe negative effect on patients’ quality of life [1] and a strong negative financial impact on health care budgets [2, 3]. Therefore, all efforts to reduce infection rates by improvement of prophylactic measures should be undertaken including antimicrobial strategies for implants.

Several alternatives have been developed as implant coatings to prevent biofilm formation: gentamicin, povidone-iodine, or silver [3]. For antibiotic-based technologies, there is a potential risk to enhance the emergence of antibiotic-resistance strains [3]. In contrast, the multilevel antimicrobial mode of silver ensures that resistance cannot be easily acquired by single-point mutations [4]. Silver exhibits significant benefits compared to antibiotics as an implant coating, including the lack of clinically relevant silver resistance of bacteria, the broad antimicrobial activity against almost all types of bacteria and fungi, and its good biocompatibility that has already been published in the context with coatings from orthopedic megaendoprostheses [5–8]. The benefits of silver for implant coatings for orthopedic devices have already been established for silver-coated megaendoprostheses in orthopedic tumor surgery with good biocompatibility [5–7, 9] and significant reduction of infection rates in a case-control study [7].

A surface modification based on a plasma electrolyte oxidation (PEO) process with the release of silver ions and antimicrobial properties for locking plates (*aap* Implantate AG, Berlin, Germany) has recently shown good biocompatibility without negative effects on fracture healing [10]. This technology is intended to use silver as a prophylactic antimicrobial agent to reduce the risk for biofilm formation on the implant surface to improve infection prophylaxis for implant-associated infections in fracture patients.

There is currently no silver-based technology commercially available for the prophylactic use in fracture patients and to the authors’ best knowledge, no study protocol in this context has been published before in the literature.

### Objectives {7}

The primary objective of this study is to evaluate the non-inferiority of the silver-coated implants compared to the uncoated implants. Furthermore, as a secondary objective, the clinical benefit of the silver-coated implants in comparison with the uncoated implants should be demonstrated.

### Trial design {8}

This is a 1:1-randomized, controlled, subject and observer-blinded multi-center non-inferiority trial.

## Methods: Participants, interventions, and outcomes

### Study setting {9}

The study is carried out at up to 20 study sites in Germany, with the following having received ethics committee approval to date:

Universitätsklinik Regensburg, Universitätsklinikum Gießen-Marburg, Agaplesion Bethesda Krankenhaus Wuppertal, Charité Universitätsmedizin Berlin, Universitätsklinikum Rostock, Universitätsklinikum Münster, LMU Klinikum der Universität München, Universitätsklinikum Dresden, Universitätsklinikum Homburg-Saar, Unfallkrankenhaus Berlin, Universitätsklinikum Bonn.

### Eligibility criteria {10}

After giving informed consent, Patients older than 18 years who suffer from fractures of the distal tibia (AO type 43 injuries) will be enrolled in the study. The fractures must be either closed with soft tissue damage of grade 1 or grade 2 according to Tscherne Oestern classification [11, 12] or open with soft tissue damage of type I, type II, type IIIA, and type IIIB fractures according to Gustilo-Anderson classification [13, 14] and must be confirmed with radiographic images. Patients with fibula fractures may also be included but must have a fracture of the distal tibia as described before. For these subjects, the fibula fracture will also be treated with coated or uncoated study devices as randomized.

If the patient has a known allergy to silver or any components of the device or has already an implanted silver-coated device inclusion will not be possible.

### Who will take informed consent? {26a}

Patients coming to the study site will be assessed by the treating orthopedic surgeon for potential participation in the study and will be informed in oral and written form by an investigator about the clinical trial. After consent, the patient will be screened for eligibility to participate based on the abovementioned criteria.

### Additional consent provisions for collection and use of participant data and biological specimens {26b}

No additional consent provisions will be obtained. As part of the informed consent form, the permitted handling of the patients’ data according to the General Data Protection Regulation (GDPR) following EU law and applicable national regulations is explained extensively. No biological specimens will be collected.

## Interventions

### Explanation for the choice of comparators {6b}

The summary description and intended purpose of the investigational device are the same as that of the comparator device. The investigational device differs from the comparator device in that its surface which has been modified by the addition of an antibacterial coating by integrating silver agglomerates using a proprietary Plasma Electrolytic Oxidation (PEO) process. Therefore, the comparator device will allow for the safety and clinical benefit of the modified surface to be assessed.

### Intervention description {11a}

Regardless of whether the patient receives the Investigational Device or the Comparator, the study schedule is identical (see Table 2). Each patient will have seven study-related visits.

#### Screening and enrolment

After the patient gave consent, a preoperative baseline for all collected assessments in connection with the defined endpoints will be done. Attention must be paid to the baseline concentration of silver in the blood, since external factors (i.e., working in a photo development laboratory) can have an influence that could falsify the study results if this is not taken into account.

#### Implantation

On the day of the implantation the patient will be randomized to one of the two treatment groups. No specifications are made by the clinical investigation plan for implantation and subsequent care. This lies in the assessment of the treating physician. The first sample for silver level analysis after implantation must be done 24 h (± 12 h) after wound closure. If a drainage has been applied to the patient, a sample of the wound fluid will be collected 24 h (± 12 h) and 48 h (± 12 h) after wound closure to be able to compare the expected high silver concentration on the local area around the implant to the silver concentration in the blood.

#### Follow-up Visits

At the five follow-up (FU) visits of up to a year the patient will be accompanied in his or her healing process and all assessments for the defined endpoints will be made. The point of study termination is defined as the date of 12-month FU for patients with regular study termination. Following completion of the 12-month FU visit, the subject will be treated according to the standard of care practice of the treating surgeon.

### Criteria for discontinuing or modifying allocated interventions {11b}

At any time and without any reason, patients can withdraw from the clinical trial without consequences. Because of compliance reasons, the investigator can end the patient’s participation. The collected data of patients who did not end the trial per protocol will be part of the Safety Analysis Set.

### Strategies to improve adherence to interventions {11c}

Surgeons will be trained in handling of the implants to avoid any issues with the wrong use of the medical device. The study sites will be closely monitored on-site and remote by a clinical research associate (CRA). The CRA will check the training records of the study team and verify the source data. In the course of the clinical trial, an unblinded CRA will visit the study site for implant accountability, counting the implant packaging, and reviewing the device deliveries.

### Relevant concomitant care permitted or prohibited during the trial {11d}

No concomitant care and interventions are prohibited during the trial.

### Provisions for post-trial care {30}

Following completion of the 12-month FU visit, the subject will be treated according to the standard of care practice of the treating surgeon. The Sponsor will maintain an adequate insurance policy covering damages arising out of the clinical trial. This insurance covers the subjects with respect to the risks involved in this study according to the clinical investigational plan.

### Outcomes {12}

Since this clinical trial should demonstrate the non-inferiority of the silver-coated implants the primary outcome is the comparison of predefined AADEs of patients treated with the investigational product and patients who received the comparator. A list of AADEs was defined based on experiences with the non-coated implants (see Table 1). Other outcomes of interest are:

- To investigate the proportion of subjects with device-related infections occurring after successful implantation of the study device and end of the 12-month FU and compare the rate between the treatment arms.
- To investigate fracture healing assessed by local and central reviewer and compare the rate of completely healed subjects between treatment arms.
- To investigate the number of hospitalizations occurring in the first 12 months post-implantation and the nights spent in hospital and compare the numbers between treatment arms.
- To investigate the change in American Orthopaedic Foot and Ankle Score (AOFAS) at each FU visit and compare endpoint between the treatment arms.
- To investigate the change in average pain at rest at each FU visit and compare endpoint between the treatment arms.
- To investigate the change in the disability rating index at each FU visit and compare endpoint between the treatment arms.
- To investigate all items assessed in the EQ-5D-5L questionnaire.
- To investigate the proportion of subjects with full weight bearing at each FU visit and compare this endpoint between the treatment arms.
- To investigate the change in silver serum levels at each scheduled FU visit and compare endpoint between the treatment arms. The change in silver serum level is defined as the difference between the respective silver level at the respective FU and the silver level at Screening/Enrollment Visit.
- To investigate the proportion of subjects with Treatment Emergent Adverse Events (TEAEs) during the 12-month FU and compare the rate between treatment arms. A TEAE is considered as any AE observed during the 12-month FU which occurred after the start of the implantation surgery.

**Table 1:** List of anticipated adverse device effects

	Incidence proportion
Device-related events	
Implant loosening	
Implant failure (e.g., plate or screw breakage)	3% [15], 5% [16]
Local Argyria^a^	23% [17]
Delayed healing/union	2.5% [18]
Procedure-related events	
Non-union	3.7% [19], 6% [15]
Wound infection	
Superficial infection	17% [15]
Deep infection	19% [20]
Malunion	6% [15], 9.5% [21], 10% [18]
Pain	25% [19], 79% [9], 87% [20]
Hematoma	
Lesion of neurovascular structures	1.1% [9] (neuropraxia)
Post-traumatic arthritis	
Shortening of the fibula	
Compartment syndrome	1.1% [9]
Functional impairment	52% [21], 66% [9]
Amputation	2% [15]
Local discomfort	10% [18]

^a^Argyria was reported in implants different from internal fixator plates and with a different coating technique. Due to the much lower systemic exposure level that can be caused by the investigational device the actual incidence is expected to be considerably lower

### Participant timeline {13}

The participant timeline is shown in Table 2.

**Table 2:** Study schedule. Assessment windows will be as follows: screening/enrollment visit (day −21 to day 0), implantation (day 0), 1-week FU visit (7 ± 3 days), 6-week FU (42 ± 7 days), 3-month FU (90 ± 14 days), 6-month FU (182 ± 14 days), 12-month FU (365 ± 30 days)

Investigations	Enrollment	Implantation	1-week	6-week	3-month	6-month	12-month
Informed consent	X						
Randomization		X					
Implantation		X					
Adverse event reporting	X	X	X	X	X	X	X
Local Argyria	X		X	X	X	X	X
Assessment of Infection	X	X	X	X	X	X	X
Blood serum (silver levels)	X	X	X	X	X	X	X
Wound fluid (silver levels)		X					
Radiological assessment (X-ray)	X		X	X	X	X	X
AOFAS score	X		X	X	X	X	X
Questionnaires: VAS (pain), DRI, EQ-5D-5L	X		X	X	X	X	X
Assessment of weight bearing	X		X	X	X	X	X

### Sample size {14}

The sample size calculation is based on the number of expected AADE of uncoated system compared to the coated system. A one-sided test at the 5% significance level and 80% power requires 96 subjects successfully implanted with either system to detect non-inferiority at a margin of 10%, i.e. an overall number of 192 subjects with successfully implanted study devices. Accounting for a 15% drop–out rate due to screening failures and surgical failures a total sample size of 226 screened subjects is considered sufficient to achieve the primary objective of the study.

### Recruitment {15}

Patients with fractures of the distal tibia will be recruited at up to 20 German study sites.

## Assignment of interventions: allocation

### Sequence generation {16a}

Treatment allocation to coated or uncoated investigational device will be randomized in 1:1 ratio. With a stratification by site and by severity of the soft tissue lesion [3 groups: (1) closed and Gustilo-Anderson type I open; (2) Gustilo-Anderson type II open; (3) Gustilo-Anderson type III open] [13, 14]^.^

### Concealment mechanism {16b}

Randomization is performed via the eCRF by the unblinded surgeon on the day of the implantation. Blinded study team members or the sponsor cannot see the patient's treatment allocation of the patient in the eCRF.

### Implementation {16c}

On the day of implantation, the surgeon will use the randomization functionalization of the eCRF for treatment allocation.

## Assignment of interventions: Blinding

### Who will be blinded {17a}

The implanting surgeon and staff involved in the surgery or handling of the investigational device will be unblinded to treatment allocation because the difference between coated and uncoated implants is visible by eye. The subject will be blinded to treatment allocation. The blinded evaluator (medical assessor) will be blinded to treatment allocation and responsible for treatment decisions and assessment of key study endpoints. Sponsor, study management, and statistical teams in addition to adjudication board and central laboratory will also be blinded.

### Procedure for unblinding if needed {17b}

If unblinding is necessary, the investigator can unblind a patient’s treatment allocation in the eCRF. Before unblinding, the investigator has to give the reason for unblinding on an extra page on the eCRF and is informed that unblinding should only happen if definitely necessary according to the opinion of the investigator.

## Data collection and management

### Plans for assessment and collection of outcomes {18a}

On site, data will be derived from patient records and collected with an electronic Case Report Form (eCRF) which is Good Clinical Practice (GCP) Compliant. At the study visits, patients will be examined by an investigator and questionnaires will be answered. Blood samples for safety data will be performed at the local laboratories, whereas blood and wound fluid samples for silver analysis will be performed at a central laboratory at the end of the study. The radiographic data and photographs of the soft tissue acquired during the study will be uploaded to the eCRF.

### Plans to promote participant retention and complete follow-up {18b}

The patients will be informed extensively about the study set-up and the requirements during the recruitment phase. It will be stressed that participation in all follow-up visits is important for the success of the clinical trial. Nevertheless, patients are allowed to withdraw from the study at any time without any reason or explanation. Patients will be asked to do at least a close-out visit after withdrawing their consent.

Patients who deviate from the clinical investigational plan will be followed up as all the other patients to ensure their safety.

### Data management {19}

Patient data, answered questionnaires, radiographic data, and photographs of the soft tissue will be collected with an eCRF. Informed consent forms will be stored within the hospitals in a locked room. All (S)AEs and protocol deviations will be documented and reported via the eCRF. The eCRF has an audit trail system to document all changes made to the data. Source data will remain at the study sites. All research data will be archived for at least 15 years after the end of the clinical trial.

### Confidentiality {27}

After signing the informed consent form, the subject will receive a study identification code. The identification code list will only be available to the study team at site and stored in the ISF. All data entered in the eCRF will only be associated with the study identification code. Radiographic images will be blackened if necessary. Only anonymized data will be reported in publications.

### Plans for collection, laboratory evaluation, and storage of biological specimens for genetic or molecular analysis in this trial/future use {33}

Not applicable, no leftover material will be stored.

## Statistical methods

### Statistical methods for primary and secondary outcomes {20a}

All statistical calculations will be made in SAS. A statistical analysis plan will be developed and finalized before data base lock and unmasking describing in detail the planned statistical analysis.

All study endpoints of this study will be summarized using descriptive summary statistics, i.e., arithmetic mean, standard deviation, minimum, median, and maximum for quantitative variables by treatment group. For qualitative variables, absolute and relative frequencies will be reported. For event-based variables (e.g., AEs) the number of events as well as the incidences (i.e., number and percentage of affected subjects) will be provided.

The primary null hypothesis that will be tested is that the difference in proportion of subjects who have at least one predefined AADE (between investigational coated device and comparator (uncoated)) is greater than or equal to 0.1 (i.e., the alternative hypothesis is successfully met if the upper bound of a two-sided 90% CI is less than +0.1).

### Interim analyses {21b}

No interim analysis for this clinical trial is planned.

### Methods for additional analyses (e.g., subgroup analyses) {20b}

No subgroup or other additional analyses for this clinical trial is planned.

### Methods in analysis to handle protocol non-adherence and any statistical methods to handle missing data {20c}

The study will employ four analysis sets. (1) Surgical Failures: a randomized subject is assigned to the set of surgical failures if the device could not be successfully implanted. (2) Full Analysis Set employs the intent-to-treat principle and includes all subjects randomized. The Full Analysis Set population is used for all intent-to-treat-based analyses. Subjects are analyzed as they were randomized. (3) Safety Analysis Set includes all treated subjects in the Full Analysis Set who are not Surgical Failures. The Safety Analysis Set is used for all primary and secondary analyses and will be analyzed as implanted. (4) The Per Protocol set will include all subjects in the Full Analysis Set without any major protocol violations. Deviations, which are occurring due to a safety issue (e.g., drop-out due to AE) will not be excluded from the Per Protocol set. Key analyses will be performed using the Per Protocol population if the number of per protocol patients is less than 90% of the full analysis set.

### Plans to give access to the full protocol, participant level-data, and statistical code {31c}

There are no plans to give access to the full protocol, participant level-data, or statistical code before sponsor received CE-mark for the investigational device. After investigational device got the CE-mark to be distributed on the European market, the study results will be published in a scientific journal. Then the datasets the clinical study and statistical code will be available from the corresponding author on reasonable request, as is the full protocol.

## Oversight and monitoring

### Composition of the coordinating center and trial steering committee {5d}

No Data Safety Monitoring Board has been appointed for this study. Since the investigational device and the comparator only differ in the silver coating, no additional SAEs are expected because of study treatment compared to standard treatment with the approved comparator. The blood samples for silver analysis will only be analyzed after data base lock, therefore, potential high silver concentrations in the serum could not be identified during the clinical trial. If a SAE is reported via the eCRF, a medical monitor will be alarmed and assess the SAE be severity and relationship to treatment.

The sponsor and the coordinating investigator will have biweekly telephone conferences to discuss the current events. Each primary investigator at each study site is responsible for the local organization of the trial, including recruitment, taking informed consent and collection of data. At every monitoring visit, the clinical research associate will try to discuss with at least one of the site investigators about the current events and inform the sponsor about the results of this discussion.

### Composition of the data monitoring committee, its role and reporting structure {21a}

Due to the low risk profile of the investigational product and the long experience with silver application for medical purposes no Data Safety Monitoring Board will be appointed for the study.

### Adverse event reporting and harms {22}

All adverse events and device deficiencies mentioned by the patient or observed by an investigator will be reported together with the causality via the eCRF. A list of predefined AADEs, crucial for the primary endpoint, is presented in the clinical investigation plan. SAEs will be reported according to the applicable regulations.

### Frequency and plans for auditing trial conduct {23}

The clinical data and conduct will be monitored by trained clinical research associates employed by the contract research organization. One hundred percent of the data will be monitored. First monitoring visit at a study site will be planned shortly after the first implantation. Following monitoring visits will be conducted according to the enrollment speed of the study site.

### Plans for communicating important protocol amendments to relevant parties (e.g., trial participants, ethical committees) {25}

All substantial amendments, as defined by the Medical Device Coordination Group [22], will be submitted to the ethics committee and the competent authority. Non-substantial amendments will be notified to the ethics committee and the competent authority. If an amendment concerns or affects patients in any way, they will be informed about the changes and if necessary, consent will be requested again.

### Dissemination plans {31a}

Both positive and negative results will be reported in international peer-reviewed journals.

## Discussion

### Selection of indication

The non-inferiority aspect of the clinical trial could be shown with any fracture of a patient, but as distal tibia fractures show by far the highest infection rate, this is the most appropriate indication for our study to show a potential clinical benefit for the patient in terms of improvement of infection prophylaxis by reduction of infection rated in the coated vs. the uncoated group.

### Radiological assessment (X-ray) — fracture healing

During the follow-up visit at weeks 1 and 6 and after 3-, 6-, and 12-month imaging assessment will be conducted. At each follow-up visit, images are reviewed to determine fracture healing:

- Callus bridging on three of four cortices on orthogonal radiographs
- No bridging callus on three of four cortices on orthogonal radiographs
- Signs of Malunion defined as more than 10 mm of shortening and more than 5 degrees of angulation in any plane

Based on these assessments fracture healing will be defined for each subject as:

- Complete healing is defined as bridging callus on three of four cortices on orthogonal radiographs within 6 months after implantation.
- Delayed Union is defined as failure to show bridging callus on three of four cortices on orthogonal radiographs after 6–9 months.
- Non-union is defined as failure to show bridging callus on three of four cortices on orthogonal radiographs after 12-month FU.
- Signs of malunion defined as more than 10 mm of shortening and more than 5 degrees of angulation in any plane after 12-month FU.

The last assessment for each subject will be considered for the secondary endpoint.

In order to get an independent assessment of the radiographic images, an independent adjudication board will be formed consisting of radiologists which are not participating in the clinical trial. All images will be sent to the independent adjudication board at the end of the study, where the images will be evaluated for healing.

### Wound fluid

To determine a local concentration of silver ions in the patient’s body close to the implant, a sample of the wound fluid should be collected 24 h and 48 h after implantation, if a drainage has been applied to the patient. To the best of our knowledge, no other clinical trial with this high number of subjects investigated the silver concentration in the wound fluid after the implantation of silver-coated devices.

### Market approval

For market approval of such an antibacterial coating, not only safety but also a clinical benefit by improving infection prophylaxis needs to be shown by clinical data compared to the uncoated comparator device. As the underlying infection rate of the trauma to be treated is extremely important for the calculation of the sample size and feasibility of such a study, the selection of an appropriate injury and indication is essential in this context.

### Trial status

The randomized controlled trial (RCT) recruitment and consequent implantations started in December 2021 and is planned until June 2023. Follow-up will be conducted over 12 months for each patient.

## Data Availability

The datasets used and/or analyzed during the current study will not be made available.

## Abbreviations

AADE: Anticipated Adverse Device Effects
AOFAS: American Orthopaedic Foot and Ankle Score
CRA: Clinical research associate
DRI: Disability Rating Index
eCRF: Electronic Case Report Form
FU: Follow-up
MRI: Magnetic resonance imaging
PEO: Plasma electrolyte oxidation
RCT: Randomized controlled trial
(S)AE: (Serious) Adverse Event
TEAE: Treatment Emergent Adverse Event
VAS: Visual analog scale
